# Supplementary material for: Modeling glioblastoma heterogeneity as a dynamic network of cell states
Source: Mol Syst Biol. 2021 Sep 16;17(9):e10105. doi: 10.15252/msb.202010105 (PMC8444284; doi:10.15252/msb.202010105)
Supplement: Supplementary file 5 — Source Data for Figure 3 [file MSB-17-e10105-s001.zip › Figure3A_sourcedata/GSEA_3065/nosigdb_state2.GseaPreranked.1622718419215/NB_KI_CLUSTER23.html]

Details for gene set NB\_KI\_CLUSTER23[GSEA]

|  || Dataset | state2 |
| Phenotype | NoPhenotypeAvailable |
| Upregulated in class | na\_neg |
| GeneSet | NB\_KI\_CLUSTER23 |
| Enrichment Score (ES) | -0.54581153 |
| Normalized Enrichment Score (NES) | -2.3504102 |
| Nominal p-value | 0.0 |
| FDR q-value | 0.0 |
| FWER p-Value | 0.0 |
Table: GSEA Results Summary

  

Fig 1: Enrichment plot: NB\_KI\_CLUSTER23      
 Profile of the Running ES Score & Positions of GeneSet Members on the Rank Ordered List

  

| PROBE | GENE SYMBOL | GENE\_TITLE | RANK IN GENE LIST | RANK METRIC SCORE | RUNNING ES | CORE ENRICHMENT || 1 | UBA2 |  |  | 223 | 0.268 | -0.0129 | No |
| 2 | POLR2F |  |  | 253 | 0.250 | -0.0054 | No |
| 3 | PHGDH |  |  | 271 | 0.243 | 0.0031 | No |
| 4 | GSTP1 |  |  | 306 | 0.230 | 0.0091 | No |
| 5 | HAT1 |  |  | 381 | 0.209 | 0.0099 | No |
| 6 | NAP1L1 |  |  | 387 | 0.207 | 0.0182 | No |
| 7 | RECQL |  |  | 390 | 0.206 | 0.0267 | No |
| 8 | BCL7C |  |  | 429 | 0.193 | 0.0308 | No |
| 9 | ARL2 |  |  | 449 | 0.191 | 0.0368 | No |
| 10 | DNAJA1 |  |  | 513 | 0.178 | 0.0375 | No |
| 11 | CTNNAL1 |  |  | 532 | 0.176 | 0.0430 | No |
| 12 | SEC61B |  |  | 812 | 0.137 | 0.0185 | No |
| 13 | SNX3 |  |  | 823 | 0.136 | 0.0232 | No |
| 14 | UGP2 |  |  | 851 | 0.132 | 0.0259 | No |
| 15 | C8orf59 |  |  | 1042 | 0.116 | 0.0101 | No |
| 16 | SNX7 |  |  | 1114 | 0.111 | 0.0071 | No |
| 17 | MGST3 |  |  | 1210 | 0.104 | 0.0012 | No |
| 18 | RDX |  |  | 1214 | 0.104 | 0.0053 | No |
| 19 | ATOX1 |  |  | 1246 | 0.102 | 0.0062 | No |
| 20 | MTLN |  |  | 1307 | 0.098 | 0.0038 | No |
| 21 | UAP1 |  |  | 1401 | 0.092 | -0.0024 | No |
| 22 | FEZ1 |  |  | 1543 | 0.084 | -0.0141 | No |
| 23 | DNPH1 |  |  | 1619 | 0.081 | -0.0189 | No |
| 24 | NQO2 |  |  | 1697 | 0.077 | -0.0240 | No |
| 25 | PRDX1 |  |  | 1763 | 0.074 | -0.0279 | No |
| 26 | GADD45A |  |  | 1791 | 0.073 | -0.0277 | No |
| 27 | SPTSSA |  |  | 1794 | 0.073 | -0.0249 | No |
| 28 | PKIG |  |  | 1797 | 0.073 | -0.0220 | No |
| 29 | TJP1 |  |  | 1811 | 0.072 | -0.0204 | No |
| 30 | FKBP1A |  |  | 1920 | 0.067 | -0.0292 | No |
| 31 | ZMYND11 |  |  | 2056 | 0.062 | -0.0413 | No |
| 32 | ROCK2 |  |  | 2074 | 0.062 | -0.0405 | No |
| 33 | TCEAL9 |  |  | 2086 | 0.062 | -0.0391 | No |
| 34 | PEA15 |  |  | 2161 | 0.059 | -0.0446 | No |
| 35 | TULP3 |  |  | 2170 | 0.058 | -0.0430 | No |
| 36 | MID1IP1 |  |  | 2313 | 0.053 | -0.0562 | No |
| 37 | ADD3 |  |  | 2401 | 0.051 | -0.0635 | No |
| 38 | SRGAP2B |  |  | 2503 | 0.047 | -0.0725 | No |
| 39 | SEPT2 |  |  | 2582 | 0.045 | -0.0791 | No |
| 40 | FHL1 |  |  | 2686 | 0.042 | -0.0885 | No |
| 41 | UBE2Q2 |  |  | 2759 | 0.040 | -0.0946 | No |
| 42 | UGDH |  |  | 2897 | 0.036 | -0.1080 | No |
| 43 | SP1 |  |  | 2960 | 0.035 | -0.1132 | No |
| 44 | PDLIM7 |  |  | 3015 | 0.033 | -0.1177 | No |
| 45 | CRIP2 |  |  | 3028 | 0.033 | -0.1176 | No |
| 46 | NSRP1 |  |  | 3175 | 0.030 | -0.1322 | No |
| 47 | CNP |  |  | 3251 | 0.028 | -0.1391 | No |
| 48 | VKORC1 |  |  | 3270 | 0.028 | -0.1399 | No |
| 49 | AKAP7 |  |  | 3313 | 0.027 | -0.1433 | No |
| 50 | SBDS |  |  | 3316 | 0.027 | -0.1424 | No |
| 51 | RTL8C |  |  | 3350 | 0.027 | -0.1448 | No |
| 52 | PARVA |  |  | 3378 | 0.026 | -0.1466 | No |
| 53 | SPART |  |  | 3454 | 0.025 | -0.1537 | No |
| 54 | TP53 |  |  | 3495 | 0.024 | -0.1571 | No |
| 55 | WDFY1 |  |  | 3564 | 0.023 | -0.1635 | No |
| 56 | AMOTL1 |  |  | 3703 | 0.020 | -0.1776 | No |
| 57 | VGLL4 |  |  | 3708 | 0.020 | -0.1772 | No |
| 58 | SRGAP2C |  |  | 3766 | 0.019 | -0.1826 | No |
| 59 | SLC9A3R2 |  |  | 3914 | 0.017 | -0.1979 | No |
| 60 | OAT |  |  | 4004 | 0.015 | -0.2069 | No |
| 61 | ARHGEF12 |  |  | 4018 | 0.015 | -0.2077 | No |
| 62 | SPATA6 |  |  | 4032 | 0.015 | -0.2084 | No |
| 63 | MTIF3 |  |  | 4052 | 0.015 | -0.2099 | No |
| 64 | OSBPL1A |  |  | 4105 | 0.014 | -0.2149 | No |
| 65 | PELO |  |  | 4170 | 0.013 | -0.2214 | No |
| 66 | FCHO2 |  |  | 4247 | 0.011 | -0.2292 | No |
| 67 | FAM229B |  |  | 4254 | 0.011 | -0.2293 | No |
| 68 | IMMP2L |  |  | 4298 | 0.010 | -0.2336 | No |
| 69 | LINC01116 |  |  | 4314 | 0.010 | -0.2348 | No |
| 70 | DUSP14 |  |  | 4320 | 0.010 | -0.2349 | No |
| 71 | PTGR1 |  |  | 4343 | 0.010 | -0.2369 | No |
| 72 | NFIB |  |  | 4378 | 0.009 | -0.2402 | No |
| 73 | SECISBP2L |  |  | 4397 | 0.009 | -0.2418 | No |
| 74 | ASXL1 |  |  | 4503 | 0.007 | -0.2529 | No |
| 75 | WARS |  |  | 4517 | 0.007 | -0.2540 | No |
| 76 | AKR1A1 |  |  | 4532 | 0.007 | -0.2553 | No |
| 77 | SEPT11 |  |  | 4584 | 0.006 | -0.2605 | No |
| 78 | PTPN12 |  |  | 4593 | 0.006 | -0.2612 | No |
| 79 | PPP1R12B |  |  | 4642 | 0.005 | -0.2662 | No |
| 80 | CRYBG3 |  |  | 4820 | 0.002 | -0.2853 | No |
| 81 | TPPP3 |  |  | 4829 | 0.002 | -0.2861 | No |
| 82 | SAV1 |  |  | 4846 | 0.002 | -0.2878 | No |
| 83 | GTDC1 |  |  | 4854 | 0.002 | -0.2885 | No |
| 84 | SOS2 |  |  | 4902 | 0.001 | -0.2935 | No |
| 85 | CLMN |  |  | 4912 | 0.001 | -0.2945 | No |
| 86 | ROBO3 |  |  | 4933 | 0.001 | -0.2966 | No |
| 87 | DDAH2 |  |  | 5011 | -0.000 | -0.3050 | No |
| 88 | HSPA1B |  |  | 5070 | -0.001 | -0.3112 | No |
| 89 | TAGLN2 |  |  | 5102 | -0.002 | -0.3145 | No |
| 90 | OPTN |  |  | 5182 | -0.003 | -0.3230 | No |
| 91 | TCF7L2 |  |  | 5199 | -0.003 | -0.3246 | No |
| 92 | MYO1C |  |  | 5468 | -0.008 | -0.3534 | No |
| 93 | FAM114A1 |  |  | 5495 | -0.008 | -0.3559 | No |
| 94 | CHN1 |  |  | 5606 | -0.010 | -0.3674 | No |
| 95 | HCFC1R1 |  |  | 5621 | -0.010 | -0.3685 | No |
| 96 | GAB1 |  |  | 5722 | -0.012 | -0.3789 | No |
| 97 | ANKH |  |  | 5757 | -0.012 | -0.3820 | No |
| 98 | ESF1 |  |  | 6012 | -0.017 | -0.4089 | No |
| 99 | CCDC50 |  |  | 6021 | -0.017 | -0.4091 | No |
| 100 | EMC2 |  |  | 6312 | -0.022 | -0.4397 | No |
| 101 | ARHGAP1 |  |  | 6345 | -0.022 | -0.4422 | No |
| 102 | DTWD1 |  |  | 6353 | -0.023 | -0.4420 | No |
| 103 | MAP4 |  |  | 6400 | -0.024 | -0.4460 | No |
| 104 | CTTN |  |  | 6421 | -0.024 | -0.4472 | No |
| 105 | CLOCK |  |  | 6429 | -0.024 | -0.4469 | No |
| 106 | DDIT3 |  |  | 6433 | -0.024 | -0.4462 | No |
| 107 | CYSTM1 |  |  | 6438 | -0.024 | -0.4456 | No |
| 108 | NES |  |  | 6455 | -0.025 | -0.4463 | No |
| 109 | SERPINB6 |  |  | 6465 | -0.025 | -0.4463 | No |
| 110 | AKAP12 |  |  | 6471 | -0.025 | -0.4458 | No |
| 111 | EPHX1 |  |  | 6547 | -0.026 | -0.4528 | No |
| 112 | GJC1 |  |  | 6648 | -0.028 | -0.4625 | No |
| 113 | DLGAP4 |  |  | 6673 | -0.029 | -0.4639 | No |
| 114 | SMIM14 |  |  | 6812 | -0.032 | -0.4775 | No |
| 115 | CTDSP2 |  |  | 6841 | -0.032 | -0.4792 | No |
| 116 | MBNL2 |  |  | 6870 | -0.033 | -0.4808 | No |
| 117 | RECK |  |  | 6921 | -0.035 | -0.4848 | No |
| 118 | RND3 |  |  | 6928 | -0.035 | -0.4840 | No |
| 119 | CTNND1 |  |  | 6998 | -0.036 | -0.4899 | No |
| 120 | MANF |  |  | 7013 | -0.037 | -0.4899 | No |
| 121 | TCEAL4 |  |  | 7014 | -0.037 | -0.4883 | No |
| 122 | FKBP7 |  |  | 7069 | -0.038 | -0.4926 | No |
| 123 | ARHGAP12 |  |  | 7074 | -0.038 | -0.4914 | No |
| 124 | FAM210B |  |  | 7076 | -0.038 | -0.4899 | No |
| 125 | TMEM87A |  |  | 7231 | -0.043 | -0.5048 | No |
| 126 | TMEM263 |  |  | 7263 | -0.044 | -0.5063 | No |
| 127 | CTNNA1 |  |  | 7279 | -0.044 | -0.5061 | No |
| 128 | KDSR |  |  | 7329 | -0.045 | -0.5095 | No |
| 129 | MPG |  |  | 7337 | -0.046 | -0.5083 | No |
| 130 | MPRIP |  |  | 7372 | -0.047 | -0.5100 | No |
| 131 | FAM234A |  |  | 7386 | -0.047 | -0.5094 | No |
| 132 | CDKN1C |  |  | 7524 | -0.051 | -0.5222 | No |
| 133 | SEC23A |  |  | 7526 | -0.051 | -0.5201 | No |
| 134 | NDRG1 |  |  | 7538 | -0.051 | -0.5191 | No |
| 135 | EVI5 |  |  | 7574 | -0.052 | -0.5207 | No |
| 136 | ACADVL |  |  | 7628 | -0.054 | -0.5242 | No |
| 137 | GRAMD1A |  |  | 7636 | -0.054 | -0.5226 | No |
| 138 | CNKSR3 |  |  | 7638 | -0.054 | -0.5204 | No |
| 139 | PDLIM5 |  |  | 7663 | -0.055 | -0.5207 | No |
| 140 | RBMS1 |  |  | 7667 | -0.055 | -0.5187 | No |
| 141 | ANKRD10 |  |  | 7760 | -0.058 | -0.5262 | No |
| 142 | ERLEC1 |  |  | 7799 | -0.060 | -0.5278 | No |
| 143 | FADS3 |  |  | 7807 | -0.060 | -0.5260 | No |
| 144 | ARMCX2 |  |  | 7832 | -0.061 | -0.5261 | No |
| 145 | SIPA1L2 |  |  | 7837 | -0.061 | -0.5239 | No |
| 146 | DYNC2H1 |  |  | 7929 | -0.064 | -0.5311 | No |
| 147 | SLC16A4 |  |  | 7964 | -0.066 | -0.5320 | No |
| 148 | DMD |  |  | 7987 | -0.067 | -0.5316 | No |
| 149 | PKD2 |  |  | 7992 | -0.067 | -0.5292 | No |
| 150 | ABL2 |  |  | 7999 | -0.067 | -0.5270 | No |
| 151 | RASAL2 |  |  | 8072 | -0.070 | -0.5318 | No |
| 152 | EXT2 |  |  | 8078 | -0.070 | -0.5294 | No |
| 153 | BMP1 |  |  | 8090 | -0.071 | -0.5276 | No |
| 154 | SLC39A14 |  |  | 8119 | -0.072 | -0.5276 | No |
| 155 | RNF24 |  |  | 8166 | -0.075 | -0.5294 | No |
| 156 | PTEN |  |  | 8202 | -0.077 | -0.5299 | No |
| 157 | SH3BP5 |  |  | 8247 | -0.080 | -0.5313 | No |
| 158 | NUCB2 |  |  | 8258 | -0.080 | -0.5290 | No |
| 159 | UFL1 |  |  | 8281 | -0.082 | -0.5279 | No |
| 160 | SHISA5 |  |  | 8318 | -0.084 | -0.5283 | No |
| 161 | CKAP4 |  |  | 8425 | -0.091 | -0.5359 | No |
| 162 | CLN5 |  |  | 8453 | -0.093 | -0.5349 | No |
| 163 | SOX5 |  |  | 8554 | -0.100 | -0.5416 | Yes |
| 164 | SYNE1 |  |  | 8557 | -0.100 | -0.5375 | Yes |
| 165 | AP1S2 |  |  | 8570 | -0.101 | -0.5345 | Yes |
| 166 | MAGED2 |  |  | 8572 | -0.101 | -0.5303 | Yes |
| 167 | LTBP3 |  |  | 8583 | -0.102 | -0.5271 | Yes |
| 168 | TMED1 |  |  | 8590 | -0.102 | -0.5234 | Yes |
| 169 | CERCAM |  |  | 8593 | -0.102 | -0.5193 | Yes |
| 170 | ITM2C |  |  | 8600 | -0.103 | -0.5155 | Yes |
| 171 | PHLDB1 |  |  | 8610 | -0.104 | -0.5121 | Yes |
| 172 | FBLIM1 |  |  | 8629 | -0.105 | -0.5096 | Yes |
| 173 | CTSL |  |  | 8637 | -0.106 | -0.5059 | Yes |
| 174 | IMPAD1 |  |  | 8643 | -0.106 | -0.5019 | Yes |
| 175 | ST5 |  |  | 8647 | -0.107 | -0.4977 | Yes |
| 176 | ITPRID2 |  |  | 8651 | -0.107 | -0.4935 | Yes |
| 177 | SCPEP1 |  |  | 8654 | -0.107 | -0.4891 | Yes |
| 178 | RGS3 |  |  | 8684 | -0.110 | -0.4876 | Yes |
| 179 | LEPROT |  |  | 8698 | -0.111 | -0.4843 | Yes |
| 180 | QSOX1 |  |  | 8731 | -0.114 | -0.4830 | Yes |
| 181 | SMPD1 |  |  | 8740 | -0.115 | -0.4789 | Yes |
| 182 | SEL1L |  |  | 8742 | -0.115 | -0.4741 | Yes |
| 183 | ENC1 |  |  | 8759 | -0.117 | -0.4709 | Yes |
| 184 | P3H1 |  |  | 8767 | -0.118 | -0.4667 | Yes |
| 185 | BMPR2 |  |  | 8774 | -0.119 | -0.4623 | Yes |
| 186 | SUMF2 |  |  | 8780 | -0.120 | -0.4577 | Yes |
| 187 | CTSF |  |  | 8795 | -0.122 | -0.4541 | Yes |
| 188 | TPST1 |  |  | 8811 | -0.124 | -0.4505 | Yes |
| 189 | SYNGR1 |  |  | 8812 | -0.124 | -0.4452 | Yes |
| 190 | TMEM30A |  |  | 8814 | -0.124 | -0.4400 | Yes |
| 191 | ASPH |  |  | 8843 | -0.128 | -0.4376 | Yes |
| 192 | SAT2 |  |  | 8870 | -0.133 | -0.4348 | Yes |
| 193 | HSD17B12 |  |  | 8878 | -0.133 | -0.4300 | Yes |
| 194 | FKBP2 |  |  | 8892 | -0.134 | -0.4257 | Yes |
| 195 | PDIA4 |  |  | 8894 | -0.134 | -0.4201 | Yes |
| 196 | SLCO3A1 |  |  | 8895 | -0.134 | -0.4144 | Yes |
| 197 | FRMD4A |  |  | 8896 | -0.135 | -0.4087 | Yes |
| 198 | DNAJC10 |  |  | 8908 | -0.137 | -0.4041 | Yes |
| 199 | CNPY2 |  |  | 8913 | -0.137 | -0.3987 | Yes |
| 200 | TMEM67 |  |  | 8933 | -0.139 | -0.3949 | Yes |
| 201 | C9orf3 |  |  | 8953 | -0.142 | -0.3909 | Yes |
| 202 | SORT1 |  |  | 8957 | -0.142 | -0.3852 | Yes |
| 203 | PGRMC1 |  |  | 8958 | -0.143 | -0.3791 | Yes |
| 204 | SGCE |  |  | 8959 | -0.143 | -0.3731 | Yes |
| 205 | SLC16A1 |  |  | 8970 | -0.145 | -0.3680 | Yes |
| 206 | PTN |  |  | 8989 | -0.147 | -0.3637 | Yes |
| 207 | TMEM43 |  |  | 9000 | -0.149 | -0.3585 | Yes |
| 208 | LRCH3 |  |  | 9010 | -0.150 | -0.3531 | Yes |
| 209 | C5orf15 |  |  | 9015 | -0.152 | -0.3471 | Yes |
| 210 | F2R |  |  | 9026 | -0.153 | -0.3417 | Yes |
| 211 | COMT |  |  | 9039 | -0.156 | -0.3364 | Yes |
| 212 | CHPT1 |  |  | 9057 | -0.159 | -0.3315 | Yes |
| 213 | PCYOX1 |  |  | 9064 | -0.160 | -0.3253 | Yes |
| 214 | CD151 |  |  | 9094 | -0.165 | -0.3215 | Yes |
| 215 | ASAH1 |  |  | 9096 | -0.166 | -0.3145 | Yes |
| 216 | LGALS3BP |  |  | 9114 | -0.169 | -0.3092 | Yes |
| 217 | FKBP9 |  |  | 9120 | -0.170 | -0.3025 | Yes |
| 218 | ATP1A1 |  |  | 9121 | -0.170 | -0.2953 | Yes |
| 219 | HEXA |  |  | 9129 | -0.172 | -0.2887 | Yes |
| 220 | SELENOS |  |  | 9135 | -0.174 | -0.2819 | Yes |
| 221 | PRDX4 |  |  | 9151 | -0.178 | -0.2760 | Yes |
| 222 | GALNT2 |  |  | 9171 | -0.182 | -0.2703 | Yes |
| 223 | HYAL2 |  |  | 9174 | -0.182 | -0.2628 | Yes |
| 224 | AGRN |  |  | 9184 | -0.185 | -0.2559 | Yes |
| 225 | SERPINE2 |  |  | 9186 | -0.186 | -0.2481 | Yes |
| 226 | FAM3C |  |  | 9190 | -0.187 | -0.2405 | Yes |
| 227 | PPFIBP1 |  |  | 9225 | -0.203 | -0.2356 | Yes |
| 228 | ITGA3 |  |  | 9226 | -0.203 | -0.2270 | Yes |
| 229 | ABHD2 |  |  | 9250 | -0.213 | -0.2204 | Yes |
| 230 | ZMAT3 |  |  | 9276 | -0.223 | -0.2137 | Yes |
| 231 | PAM |  |  | 9280 | -0.224 | -0.2045 | Yes |
| 232 | LAMB1 |  |  | 9302 | -0.231 | -0.1970 | Yes |
| 233 | TMED9 |  |  | 9305 | -0.233 | -0.1874 | Yes |
| 234 | ATP2B4 |  |  | 9314 | -0.243 | -0.1779 | Yes |
| 235 | LAMP1 |  |  | 9317 | -0.244 | -0.1678 | Yes |
| 236 | CD9 |  |  | 9324 | -0.248 | -0.1579 | Yes |
| 237 | SCARB2 |  |  | 9329 | -0.251 | -0.1477 | Yes |
| 238 | ZBTB20 |  |  | 9341 | -0.261 | -0.1378 | Yes |
| 239 | ITGB8 |  |  | 9362 | -0.294 | -0.1275 | Yes |
| 240 | ATP6AP2 |  |  | 9365 | -0.294 | -0.1152 | Yes |
| 241 | GLG1 |  |  | 9371 | -0.306 | -0.1027 | Yes |
| 242 | TIMP2 |  |  | 9379 | -0.316 | -0.0901 | Yes |
| 243 | VCL |  |  | 9405 | -0.389 | -0.0763 | Yes |
| 244 | APLP2 |  |  | 9409 | -0.408 | -0.0593 | Yes |
| 245 | APP |  |  | 9412 | -0.426 | -0.0414 | Yes |
| 246 | DST |  |  | 9422 | -0.485 | -0.0218 | Yes |
| 247 | DCBLD2 |  |  | 9431 | -0.558 | 0.0010 | Yes |
Table: GSEA details [plain text format]

  

Fig 2: NB\_KI\_CLUSTER23: Random ES distribution      
 Gene set null distribution of ES for **NB\_KI\_CLUSTER23**
